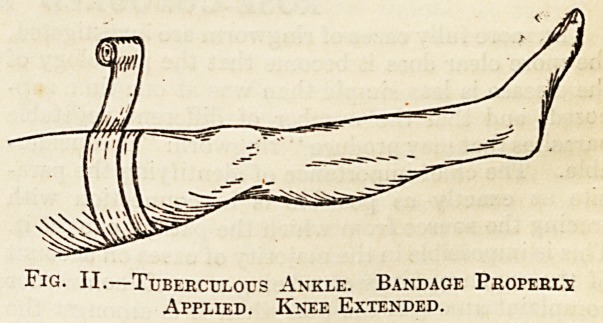# The Hyperæmic Method of Treatment—III

**Published:** 1909-02-06

**Authors:** 


### Orthopædics.

### THE HYPERÆMIC METHOD OF TREATMENT—III.

In the preceding article the treatment by hyperæmia, induced by suction apparatus or the so-called "Bier's cups," has been dealt with. It now remains to explain and illustrate the technique of the second method, in which hyperæmia is induced by means of a constricting bandage. This differs from the suction method in several important points. In the latter the reaction round the lesion is augmented by "sucking the blood towards that spot"; in other words, the hyperæmia is due mainly (though not entirely) to dilatation of the vessels and an increased flow *towards* the lesion. It is therefore essentially an arterial hyperæmia, and is the closest imitation of the natural hyperæmia, which is the result of inflammation and increased tissue reactions. In the constriction method the engorgement is produced by preventing or retarding the venous return flow. The arteries are not interfered with. They are allowed to carry their blood to the part, but that blood is in a great measure prevented from returning to the heart through the venous channels. The obstruction is not absolute, for the deeper veins are not affected by the bandage, but is largely superficial; yet in all cases it is sufficient to cause a marked degree of hyperæmia.

This method is the most valuable, and from a practical point of view the most generally useful of the three methods recognised by the Bier school—the hot-air, suction, and constriction methods. It is easily mastered, and its range of usefulness is almost unlimited. In these articles we are only concerned with orthopædic cases, but it must be borne in mind that the hyperæmic method of treatment is usefully applicable to a large variety of both medical and general surgical lesions. All inflammatory conditions, whether acute or chronic, are benefited by it.

At the same time it is useful to draw attention to the fact that, however encouragingly quick and speedy this method of treatment by hyperæmia acts in certain cases of acute inflammatory lesions, it is a method that calls for much perseverance and patience on the part of both the surgeon and the patient when it is employed in chronic cases. It is a method that rarely fails in suitable cases, but complete success with it means unlimited patience for the doctor as well as for the patient, who must persevere with it for months, and in some obstinate cases for years.

To abide by the orthopædic case as illustration, we will again assume that the patient is suffering from a tubercular foot. This time, however, there are no sinuses. The disease may have advanced very far. The tarsal and ankle joints may be involved, and there may be extensive pulpy disease, with fluctuating and tender spots readily discoverable on palpation; but there is no open wound, no ulceration, no sinus. The skin is unbroken, but there may be much pain and extensive involvement of the joint surfaces. This is a case which, before any operative interference is attempted, should be given the benefit of the hyperæmic treatment for a couple of months at least. In describing how the method is applied in such a case, it must be remembered that the description applies to all other lesions, no matter where or what they are, provided that they are in such a position that the bandage can be applied above them.

The affected limb is bared and allowed to rest on a chair or couch. The patient either sits or lies down. The bandage, which must be of thin rubber

Fig I.—Tuberculous Ankle. Bandage Badly Applied.

or garter elastic, if a proper Bier's bandage is not at hand, is carefully put round the limb as far away from the lesion as possible. Figs. I. and II. illustrate the wrong and the right method of applying it. In the first block the bandage has been put on much too low down, and the flexion of the knee joint is a further fault; in the second block the bandage is shown properly applied—that is, high up in the thigh, as far above the site of the lesion, with the leg extended and the foot resting on a couch. A

Fig. II.—Tuberculous Ankle. Bandage Properly Applied. Knee Extended.

good rule to adopt, so far as joint lesions are concerned, is to apply the bandage at least above the joint immediately above the affected articulation. Thus, in the case of a phalangeal lesion the bandage is put well above the wrist or ankle joint. In the case of the knee joint, where it is impossible to go above the hip joint, the bandage is put as high up as possible; in an elbow-joint affection the shoulder is encircled by the elastic.

Some skill is needed in applying the bandage. It should be put on at one spot only, each turn being allowed to lie immediately above its predecessor. There must be no creasing or folds in the bandage, and usually three or four turns are quite sufficient. The final turn may be put on a little tighter than the others, when it will be found to act as a stay to the whole, no knot or band being required to keep the bandage on. Where webbing bandages, which do not "grip" so well as the rubber ones, are used, they must be furnished with tags to enable them to be tied. The bandage must be put on moderately tightly—not too tightly, else it will interfere with the arterial flow and with the blood in the profunda and companion veins, and will also cause pain. When it has been put on too tightly the limb becomes dark blue, cold, and—if the bandage is allowed to remain on long enough—gangrenous. Neither must it be put on too slackly, else no hyperæmia will result. The proper degree of tightness can only be learned by experience, but the following hints may be of service to those who are trying the method for the first time:—The arteries below the bandage must always be felt pulsating well; the limb below the constriction must become reddened, the redness deepening into a slightly bluish tinge, and showing mottling here and there; it must feel warmer than the limb on the other side, and look plumper and more solid; and, lastly, the patient must not feel a sense of pain or constriction at the site where the bandage is applied. Usually, however, the patient does feel tingling or actual pain at the site of the lesion. This may be very severe in acute cases, while in chronic ones, such as the tuberculous joint cases, it may be trivial. In any case it passes off when the condition of hyperæmia is well established.

### Its Effects.

The patient can usually be taught to put on the bandage himself, but he should be closely supervised, as it is possible to do more harm with this method than with the suction cup. He should be taught to recognise the proper colour of the hyperæmic limb—neither the dark blue of the fully constricted vessels nor the redness of merely superficial stasis, but a happy medium between the two. To an experienced eye a glance at the limb is sufficient to show whether or not the bandage has been properly applied, and after treating a few cases the practitioner will easily obtain enough experience to enable him to judge the degree of constriction by the hue of the limb.

The results of this method are excellent, especially when it is used as an auxiliary to other methods. Thus in the case of tuberculous joints proper orthopædic treatment, by splints or otherwise, must be carried on at the same time that the hyperæmic method is being used. Employed in combination, judiciously, and in selected cases, it will be found to be an exceedingly valuable method of treatment; but the practitioner must not expect to work miracles by its means. He will find enough, however, to satisfy himself that it is a valuable help to him. Under it some tuberculous joints recover with astonishing rapidity and completeness, while he will obtain equally good results in cases of acute arthritis, especially in the rheumatic, gouty, and gonorrhœal varieties. The pneumococcal form is also amenable to the hyperæmic treatment, although the results, so far as pneumococcal arthritis is concerned are not brilliant. In all cases suitable operative treatment must be considered where necessary, but in tubercular cases the practitioner should not be too hasty in operating, as under the hyperæmic method very bad cases sometimes improve in an astonishing degree.

In the following articles, when dealing with special lesions, we shall give the indications for the treatment by Bier's method in more detail wherever it is applicable.

## Figures and Tables

**Fig I. f1:**
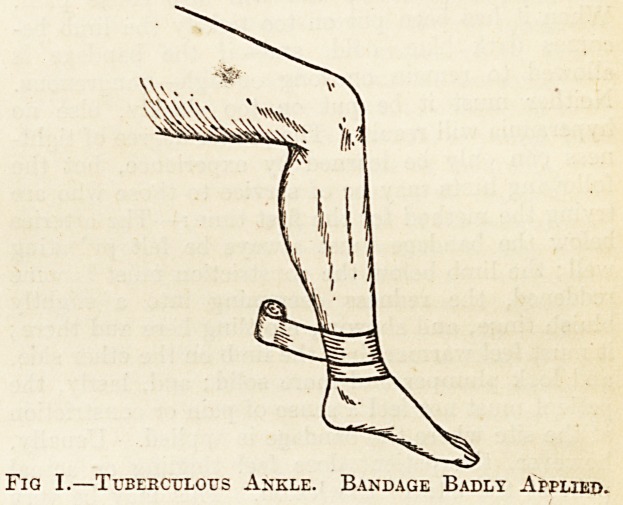


**Fig. II. f2:**